# Development and Optimization of Sildenafil Orodispersible Mini-Tablets (ODMTs) for Treatment of Pediatric Pulmonary Hypertension Using Response Surface Methodology

**DOI:** 10.3390/pharmaceutics15030923

**Published:** 2023-03-12

**Authors:** Ahmed Alalaiwe, Mohammad A. Alsenaidy, Ziyad S. Almalki, Mohamed H. Fayed

**Affiliations:** 1 Department of Pharmaceutics, College of Pharmacy, Prince Sattam Bin Abdulaziz University, Al-kharj 11942, Saudi Arabia; 2 Department of Pharmaceutics, College of Pharmacy, King Saud University, Riyadh 11451, Saudi Arabia; 3 Department of Clinical Pharmacy, College of Pharmacy, Prince Sattam Bin Abdulaziz University, Al-kharj 11942, Saudi Arabia; 4 Department of Pharmaceutics, Faculty of Pharmacy, Fayoum University, Fayoum 63514, Egypt

**Keywords:** orodispersible mini-tablets, sildenafil citrate, pediatric formulation, design-of-experiment

## Abstract

The availability of age-appropriate oral dosage forms for pediatric patients has remained a challenge. Orodispersible mini-tablets (ODMTs) are a promising delivery system for pediatric patients. The purpose of this work was the development and optimization of sildenafil ODMTs as a new dosage form for the treatment of pulmonary hypertension in children using a design-of-experiment (DoE) approach. A two-factor, three levels (3^2^) full-factorial design was employed to obtain the optimized formulation. The levels of microcrystalline cellulose (MCC; 10–40% *w*/*w*) and partially pre-gelatinized starch (PPGS; 2–10% *w*/*w*) were set as independent formulation variables. In addition, mechanical strength, disintegration time (DT), and percent drug release were set as critical quality attributes (CQAs) of sildenafil ODMTs. Further, formulation variables were optimized using the desirability function. ANOVA analysis proved that MCC and PPGS had a significant (*p* < 0.05) impact on CQAs of sildenafil ODMTs with a pronounced influence of PPGS. The optimized formulation was achieved at low (10% *w*/*w*) and high (10% *w*/*w*) levels of MCC and PPGS, respectively. The optimized sildenafil ODMTs showed crushing strength of 4.72 ± 0.34 KP, friability of 0.71 ± 0.04%, DT of 39.11 ± 1.03 s, and sildenafil release of 86.21 ± 2.41% after 30 min that achieves the USP acceptance criteria for ODMTs. Validation experiments have shown that the acceptable prediction error (<5%) indicated the robustness of the generated design. In conclusion, sildenafil ODMTs have been developed as a suitable oral formulation for the treatment of pediatric pulmonary hypertension using the fluid bed granulation process and the DoE approach.

## 1. Introduction

Pulmonary arterial hypertension (PAH) is a life-threatening disease in children [1]. Recently, clinical trials showed the successful use of phosphodiesterase type 5 inhibitors (such as sildenafil) for the treatment of PAH in children, as sildenafil is simple to administer and well tolerated in children [2]. For children with PAH, oral doses of sildenafil are as follows: (1) children of less than one year were given 0.5–1 mg/kg three times daily (TID), (2) children of more than one year and less than 20 kg were given 10 mg TID, and (3) children of more than one year and more than 20 kg were given 20 mg TID [2]. Provenza et al. succeeded in developing an oral liquid formulation of sildenafil (2 mg/mL) to be used for the treatment of PAH in children [3]. However, liquid formulations have had stability problems and a high cost of storage and transportation [4]. Therefore, the development of a pediatric oral solid dosage form is highly recommended. The World Health Organization recommends the development of age-appropriate solid dosage forms such as multi-particulates and mini-tablets as the optimum oral formulations for the pediatric population [5]. However, the availability of age-appropriate solid dosage forms for pediatric patients remains a challenge [6]. Recently, it was reported that mini-tablets with a diameter of 2–3 mm are superior to liquid dosage forms for administration to pediatric patients as they provide accurate dosing and a high degree of dose flexibility [6,7,8].

It was reported that the children at the age of 2–5 years showed no problem swallowing the placebo mini-tablets [4]. However, children below six months only swallow liquid formulations [4]. Thus, orodispersible mini-tablets (ODMTs), which rapidly and readily disintegrate in the oral cavity with a small volume of saliva, could be an appropriate alternative dosage form for pediatric patients, particularly infants and toddlers. ODMTs could be considered one of the most accepted oral delivery systems for pediatrics as the risk of choking is low because they are dispersed orally [9]. It was reported that ODMTs disintegrate in the child’s oral cavity into small particles in a few seconds (<60 s) [10]. In addition, ODMTs demonstrate high stability as the drug remains in a solid state until it is administered. Thus, ODMTs have the advantages of solid formulations in terms of stability and liquid formulations in terms of the reduced risk of suffocation during oral administration [11].

ODMTs can be prepared by the lyophilization, molding, or simple direct compression (DC) process [12]. DC is the preferred method for manufacturing ODMTs because it is easy and cost-effective [13]. However, manufacturing the mini-tablets with acceptable weight and content uniformity is a challenging task. It was reported that the risk of unacceptable content uniformity and weight variation increases as the size of the tablet and drug loading decreases [7]. One approach to reducing the risk of content uniformity at low-drug loading is to decrease the particle size of the incorporated drug. However, reducing the drug particle size may lead to poor flow of formulation powder due to increased particle cohesion as well as increased risk of segregation due to variations in particle size between drug and additives [7]. These issues may result in high variability in tablet weight and provide tablets with unacceptable content uniformity using a direct compression platform. The pharmaceutical granulation process showed advantages for overcoming these issues in terms of improved weight and content uniformity [14]. In addition, granulation using a fluid bed is the most suitable granulation method for manufacturing orodispersible tablets, as it produces highly porous granules that provide tablets with rapid disintegration and dissolution [15].

To the best of our knowledge, the development of sildenafil ODMTs using the fluid bed technique has not been reported. Thus, the aim of the present work was to design, develop, and optimize sildenafil ODMTs with acceptable quality attributes (i.e., weight uniformity, content uniformity, mechanical strength, disintegration time, and drug release) using the fluid bed granulation method and Design-of-Experiment (DoE) approach. In order to obtain stable and robust sildenafil ODMTs formulation, the relationship between formulation variables must be defined and understood. Thus, the DoE approach was applied to simultaneously examine the influence of various variables and potential interactions and to predict their combined effect on the CQAs of the product [16]. With respect to the pharmaceutical design of the experiment process, two steps are important: defining and determining the correlation between independent formulation variables and dependent responses and determining the levels of those formulation variables that can provide a better response [17].

Based on the preliminary experiments and the literature evaluation, the levels of PPGS and MCC have been chosen as the most critical formulation variables to be optimized via the DoE approach. PPGS was selected as the binder/disintegrant as it improves the compressibility and flowability of granules as well as enhances the disintegration of tablets. MCC was selected as the diluent because of its binding property. Moreover, MCC is self-disintegrating and needs a small amount of lubricant. The quality target product profile (QTPP) and CQAs for sildenafil MODTs are listed in Table 1.

## 2. Materials and Methods

### 2.1. Materials

Sildenafil citrate, D- Mannitol: Mannogem^®^, sodium saccharine, and FDA-approved vanilla flavor (gift samples from JPI Co., Riyadh, Saudi Arabia). Partially pre-gelatinized starch (PPGS): Starch 1500^®^ (gift sample from Colorcon, Dartford, UK), microcrystalline cellulose (MCC): Avicel PH 105^®^ (gift sample from FMC biopolymer, St. Louis, MO, USA). Sodium stearyl fumarate: PRUV^®^ was received from JRS pharma (Rosenberg, Germany).

### 2.2. Design of Experiments and Statistical Analysis

Development and optimization of ODMTs were performed using the DoE approach; this approach helps to recognize the significant variables and best-optimized combinations to achieve the desired quality attributes of ODMTs. The levels of MCC (X_1_; 10–40% *w*/*w*) and PPGS (X_2_; 2–10% *w*/*w*) were chosen as independent formulation parameters, and these were investigated at three levels as 3^2^ full-factorial design using Design Expert^®^ 12 software (version 12, State-ease, Inc., Minneapolis, MN, USA) to generate nine experimental runs, as listed in Table 2. To recognize the reproducibility of experiments, the run of the center point was repeated three times on different days. The responses were mean granule size (y_1_), bulk density (y_2_), granule flowability (y_3_), crushing strength (y_4_), friability (y_5_), disintegration time (y_6_), and percent drug release (y_7_) of prepared ODMTs. The goal was to maximize the mechanical properties and minimize the DT of prepared tablets to obtain the optimized formulation. The ANOVA analysis at *p* < 0.05 was performed to recognize the significant effects of independent parameters on selected responses. A second-order polynomial equation (Equation (1)) was applied to identify the relationship between independent variables and measured responses.

(1) $$Y=b_{0}+b_{1}X_{1}+b_{2}X_{2}+b_{12}X_{1}X_{2}+b_{11}X_{1}{}^{2}+b_{22}X_{2}{}^{2}$$

where Y is dependent variables; b_0_ is an intercept; b_1_ and b_2_ are regression coefficients; and X_1_ and X_2_ are the investigated independent parameters. The terms of X_1×2_ and $X_{i}{}^{2}$ (i = 1 and 2) are the interaction and quadratic effects, respectively. The present design was validated through the calculation of the prediction error (PE) using Equation (2). The PE < 5% proves the accuracy and robustness of the selected models.

(2) $$\mathrm{PE}=100\times \left(\;\frac{\mathrm{Predicted}\;\mathrm{value}-\mathrm{Experimental}\;\mathrm{value}\;}{\mathrm{Predicted}\;\mathrm{value}}\right)$$

### 2.3. Production of Granules and Tablets

Table 3 lists the composition of the investigated formulations. Granules were prepared using a fluid bed granulator (Oyster Huttlin mycromix, BOSCH Packaging Technology, Schopfheim, Germany) at a batch of 600 g for all runs. All excipients except sodium stearyl fumarate were mixed in a v-shaped blender (Erweka, Apparatebau, Germany) for 10 min at 65 rpm. The powder blend was then transferred to the fluid bed and granulated by spraying with deionized water (225 mL) using a centered top spray nozzle (diameter of 0.8 mm) at a spray rate of 3 g/min. The velocity of fluidized air was adjusted (50 m^3^/h) during the process to avoid vigorous fluidization and granules attrition. Drying was carried out in the same fluid bed at 65 °C to a value of 2% moisture content. The dried granules were taken out of the fluid bed, transferred to the v-shaped blender, and blended with sodium stearyl fumarate for a further 2 min at 40 rpm. The lubricated blend was then taken out of the mixer and loaded into the hopper of a Korsch XL 100 tablet press (Korsch Pressen, Berlin, Germany) equipped with a 3-mm standard concave, five-tip tooling at 30 rpm turret speed and compression force of 8 KN. The produced ODMTs were collected and stored in a tightly closed glass container for further characterization.

### 2.4. Granules Characterization

#### 2.4.1. Measurement of Granule Size

A laser diffraction technique using a Malvern 2000 (Malvern Instruments Co., Ltd., Malvern, UK) was applied to characterize the granule size (d_50_) of three samples for each granulation run. One bar of dispersive air pressure, 1.6 g feed quantity, and obscuration were adjusted at approximately 1%.

#### 2.4.2. Measurement of Granules Bulk and Tapped Density

Bulk density was determined by gently pouring a sample of about 40 mL into a pre-weighed 50 mL graduated cylinder. The bulk density was obtained by dividing the mass of granules (g) by the volume (mL) occupied in the cylinder. Tapped density was calculated by dividing the mass of granules (g) by the volume (mL) occupied by granules after it had been tapped for a defined period of time. Carr’s index was calculated based on bulk and tapped density data.

#### 2.4.3. Measurement of Granules Flowability

Granules flow was assessed using the angle of repose method. The test was performed according to the method mentioned in USP and our previous work [14].

### 2.5. Tablets Characterization

#### 2.5.1. Weight and Content Uniformity

The weights of prepared ODMTs (n = 20) were obtained using a digital analytical balance (Mettler-Toledo, Columbus, OH, USA). The ODMTs were weighed individually, and the average weight was calculated and compared to the acceptance value mentioned in the United States Pharmacopeia (USP). Content uniformity (CU) of the prepared ODMTs was determined according to the following procedure. Ten tablets were individually crushed, dissolved in methanol, and filtered through a 0.45 µm membrane filter. Sildenafil content was analyzed using an HPLC (HPLC, Waters 1525, Milford, MA, USA) system equipped with UV detection at 292 nm. The sample analysis was carried out according to the method described by Yi et al. with modification [18]. If all 10 tablets attained drug content within 85–115% of the target potency and Relative Standard Deviation (RSD) was less than 5.0%, the prepared ODMTs were considered uniform [6].

#### 2.5.2. Crushing Strength/Breaking Force

The crushing strength of the prepared ODMTs (n = 10) was measured in kiloponds (KP) using a tablet hardness tester (Erweka, Heusenstamm, Germany).

#### 2.5.3. Friability

Using a friability tester (Erweka, TA3R, Heusenstamm, Germany), about 6.5 g (M_1_) of ODMTs were tumbled at 25 rpm for 4 min. After tumbling, the ODMTs were collected, de-dusted, and weighed once more (M_2_). The percent loss in weight (F) was determined using Equation (3).

(3) $$F=100\times \left(\;\frac{M_{1}-M_{2}\;}{M_{2}}\right)$$

where $M_{1}$ and $M_{2}$ are the initial and the final weights of the ODMTs samples, respectively.

#### 2.5.4. Disintegration Time

The disintegration time (DT) of ODMTs (n = 6) was measured at 37 ± 0.5 °C using a fully automated disintegration tester (Erweka ZT3, Heusenstamm, Germany). The test was performed according to the method mentioned in USP and our previous work [19] with minor modifications due to the smaller size of prepared MODTs. A 30–mesh woven stainless steel wire cloth with plain square weave was affixed at the bottom of the basket assembly in place of the original 10–mesh wire cloth.

#### 2.5.5. In Vitro Dissolution Study

In vitro dissolution study (n = 6) was performed in a basket dissolution apparatus (Erweka, Heusenstamm, Germany), and the basket was rotated at 50 rpm. The test was conducted in 500 mL of phosphate buffer at 37 ± 0.5 °C (PH, 6.8 ± 0.05) to simulate saliva fluid. Two milliliters of samples were withdrawn at time intervals of 5, 10, 15, and 30 min and replaced with fresh medium. Samples were analyzed using an HPLC system equipped with a UV detection set at 292 nm. The sample analysis was carried out according to the method described by Yi et al. with modifications [18].

## 3. Results and Discussion

### 3.1. Fitting Data to the Models

**Figure 1:**
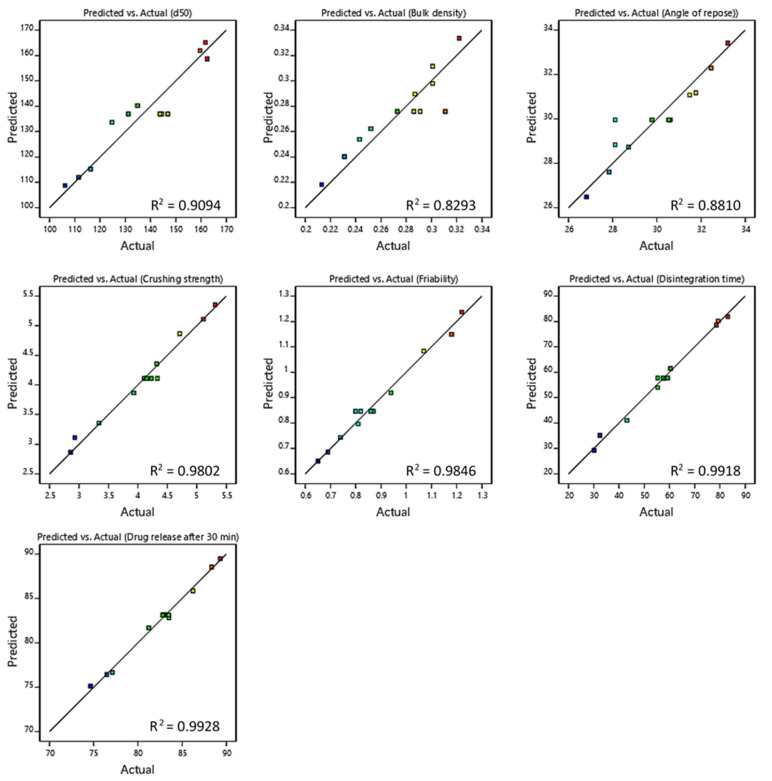
Linear correlation plot relating mean granule size, bulk density, angle of repose, crushing strength, friability, disintegration time, and drug release after 30 min between the predicted and the actual values.

Different statistical parameters, including *p*-value, R^2^, and adequate precision, were compared to recognize the best-fitting model. Table 4 lists the summary of model fitting and statistical analysis. For all suggested models, the adjusted R^2^ is in reasonable agreement with the predicted R^2^ (the difference <0.2), indicating good data fitting. In addition, *p* < 0.05 for all models proves significant model fitting. Further, the adequate precision was >4, which means that the models can be used to navigate the design space. On the other hand, Figure 1 exhibits an excellent correlation (R^2^ close to 1.0) between the predicted and the observed values, which proves a perfect model fit.

### 3.2. Influence of Independent Variables on Mean Granules Size

As shown in Table 5, the obtained mean granule size (d_50_) ranged from 106.13 ± 0.35 to 162.45 ± 0.12 µm. The influence of independent parameters on the d_50_ was explained by the empirical model of Equation (4).

(4) $$d_{50\left(\mu m\right)}=136.95+3.26{\;X}_{1}+24.97{\;X}_{2}$$

**Figure 2:**
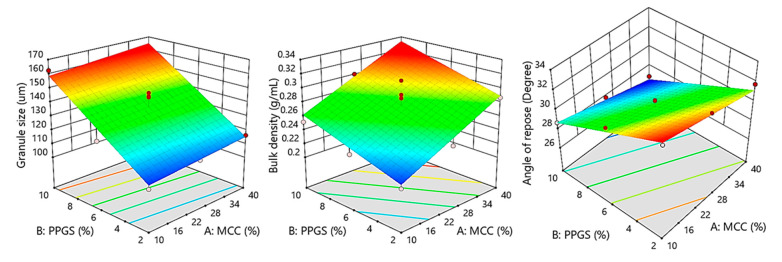
Response surface plots showing the influence of MCC (X_1_) and PPGS (X_2_) on measured granules’ responses; mean granule size (y_1_), bulk density (y_2_), and angle of repose (y_3_).

The regression analysis displayed in Table 6 demonstrates that the PPGS had a significant effect on the d_50_ (*p* < 0.0001), while the MCC did not (*p* = 0.2503). As shown by Equation (4), the significant term had a positive effect on d_50_, as proved by the positive sign of coefficient estimate (+24.97 for PPGS), illustrating that increasing the concentration of PPGS resulted in a significant increase in d_50_. This positive effect may be due to the binding effect of PPGS, which promoted the powder to stick and coalesce more readily. The response surface plot in Figure 2 demonstrates that the level of PPGS was the variable that induced the largest increase in d_50_, which was in agreement with the results reported by Alali et al. [15]. It was noticed that a 5-fold increase in the level of PPGS (2–10% *w*/*w*) resulted in a 1.5-fold increase in the d_50_.

### 3.3. Effect of Independent Variables on Granules’ Bulk Density

As depicted in Table 5, the granules’ bulk density ranged from 0.213 ± 0.014 to 0.322 ± 0.019 g/mL, which revealed that the bulk density of the obtained granules was greatly improved. The influence of independent parameters on the granules’ bulk density was demonstrated by the empirical model of Equation (5).

(5) $$\mathrm{Bulk}\;\mathrm{density}\;\left(g/\mathrm{mL}\right)=0.2759+0.0357{\;X}_{1}+0.022{\;X}_{2}$$

The regression analysis listed in Table 6 reveals that all independent parameters had significant impacts on granules’ bulk density (*p* < 0.0003 for MCC and *p* < 0.007 for PPGS). As shown by Equation (5), all the significant terms had a positive impact on granules’ bulk density based on the sign of coefficients (+0.0357 for MCC and +0.022 for PPGS), suggesting that increasing the level of these variables resulted in a higher granules’ bulk density. Figure 2 shows that a high level of MCC and PPGS would result in a higher granule density. However, the response surface plot demonstrates the pronounced influence of MCC on granules’ bulk density in a positive direction.

### 3.4. Effect of Independent Variables on Granules Flowability

As listed in Table 5, the flowability of obtained granules varied from 26.81 ± 0.392° to 33.21 ± 0.321°, which proved that the flowability of the obtained granules was significantly improved. Moreover, the results of Carr’s index are in accordance with the results of the angle of repose (Table 5). The influence of independent parameters on the granules’ flowability is demonstrated by the empirical model of Equation (6).

(6) $$\mathrm{Angle}\;\mathrm{of}\;\mathrm{repose}\;\left(\mathrm{degree}\right)=29.95-1.12{\;X}_{1}-2.35{\;X}_{2}$$

The regression analysis depicted in Table 6 demonstrates that the two parameters had significant effects on granules’ flowability (*p* < 0.0065 for MCC and *p* < 0.0001 for PPGS). Additionally, the PPGS level had the greatest effect on granules’ flowability, followed by the MCC level, with respect to the sum of square values (32.99 for PPGS and 7.55 for MCC). The negative sign of coefficients estimate in Equation (6) (−1.12 for MCC and −2.35 for PPGS) showed that the two variables had positive effects on improving granules’ flowability, integrated with the response surface plot (Figure 2); it could be noticed that granule flowability achieved its superior value with the simultaneous increase in the levels of MCC and PPGS. In contrast, granules’ flowability was lowest when both variables were at their minimum. This might be because increasing the levels of PPGS and MCC improved the granule size, which might lead to an increase in granules’ flowability. A high correlation between the angle of repose values and the granule size (r^2^ = 0.8075) was observed.

### 3.5. Weight Variability and CU of ODMTs

Obviously, maintaining low weight and CU variability for mini-tablets is a challenging task. Gupta et al. reported that a slight deviation in the weights of small-sized micro-tablets could result in a higher weight variation [7]. Interestingly, as listed in Table 7, the weight variation for each trial was observed to be in the acceptable range according to the acceptance criteria of USP. The prepared ODMTs were well within 5% of the expected weight and had an RSD of less than 2%. This proved that a consistent filling of die cavities and mass balance between the powder input and the mini-tablet output during the compression cycle would finally result in an improved weight variability of obtained ODMTs. This observation indicates the better flow properties of prepared granules. However, small variations in tablet weight could be due to small variations in the flowability and the bulk density of the obtained granules [20]. On the other hand, the CU of the obtained ODMTs ranged from 98.76 ± 2.01 to 101.56 ± 0.98%, which is well within the acceptable range according to the USP (85–115%), indicating that drug particles were uniformly distributed within the granule matrix resulting in the homogeneity of obtained granules and uniformity of dosage units. Importantly, the fluid bed granulation process produced ODMTs with better weight and CU variability compared to the mini-tablets manufactured using DC and high-shear granulation processes [7], which ultimately enhances the dosing flexibility in pediatric clinics.

### 3.6. Influence of Independent Variables on Tablet Crushing Strength and Friability

As shown in Table 7, the crushing strength of the obtained ODMTs ranged from 2.86 ± 0.61 to 5.31 ± 0.26 KP. Additionally, all ODMTs demonstrated a considerably low crushing strength, which is preferred for fast disintegration in the oral cavity. Further, variability in tablet crushing strength was observed due to reducing the tablet size. The same result was reported by Mitra et al. [6]. Therefore, a suitable selection of compression tooling is critical for the production of mini-tablets in order to reduce the variability in tablet mechanical strength. The influence of independent parameters on the crushing strength of ODMTs is demonstrated by the empirical model of Equation (7).

(7) $$\mathrm{Crushing}\;\mathrm{strength}\;\left(\mathrm{KP}\right)=4.11+0.245{\;X}_{1}+1.0{\;X}_{2}$$

**Figure 3:**
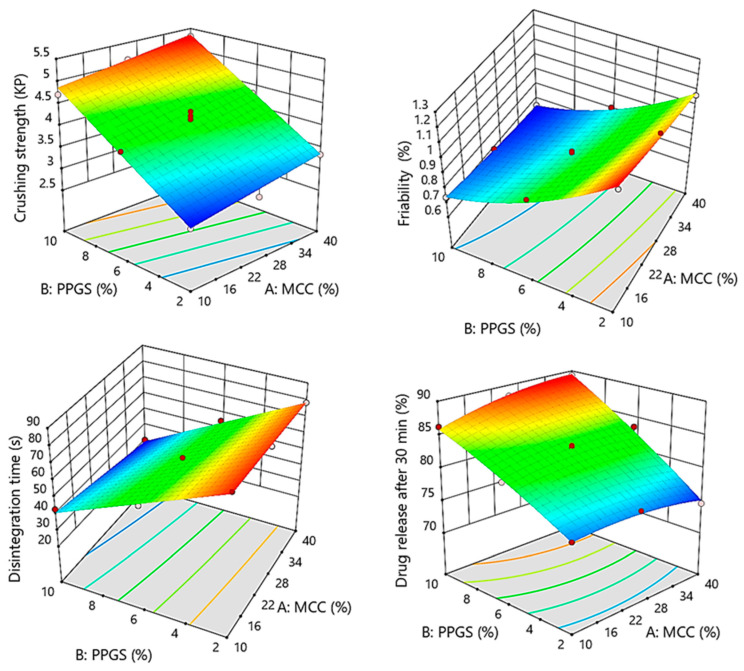
Response surface plots showing the influence of MCC (X_1_) and PPGS (X_2_) on measured MODTs responses; crushing strength (y_4_), friability (y_5_), disintegration time (y_6_), and percent release after 30 min (y_7_).

The ANOVA analysis listed in Table 8 demonstrates that all independent variables had significant effects on tablet crushing strength (*p* < 0.0007 for MCC and *p* < 0.0001 for PPGS). As shown by Equation (7), all the significant variables had a positive impact on tablet crushing strength based on the sign of coefficients estimate (+0.245 for MCC and +1.0 for PPGS), indicating that increased levels of these variables resulted in a higher crushing strength of the obtained ODMTs. This is due to the excellent compactibility of MCC at low compression pressure as well as the better binding effect of PPGS [21,22]. Combined with the response surface plot shown in Figure 3, a high level of MCC and PPGS would result in higher crushing strength, as shown in the top corner of the response surface plot. However, the response surface plot shows the predominant effect of PPGS on tablet crushing strength in a positive direction.

As listed in Table 7, the friability of obtained ODMTs varied from 0.65 ± 0.03% to 1.22 ± 0.03%. The influence of independent parameters on the friability of prepared ODMTs is explained by the empirical model of Equation (8).

(8) $$\mathrm{Friability}\;\left(\%\right)=0.8462-0.0617{\;X}_{1}-0.2317{\;X}_{2}+0.0150\;X_{1}X_{2}+0.0113\;X_{1}{}^{2}+0.0712\;X_{2}{}^{2}$$

The ANOVA analysis depicted in Table 8 reveals that both variables had significant effects on the friability of the prepared ODMTs (*p* < 0.0027 for MCC and *p* < 0.0001 for PPGS). Additionally, the PPGS level had the strongest effect on tablet friability, followed by the MCC level, with respect to the sum of square values (0.3220 for PPGS and 0.0228 for MCC). The negative sign of coefficients estimate in Equation (8) (−0.0617 for MCC and −0.2317 for PPGS) showed that the two variables had positive effects on reducing the friability of the prepared ODMTs. Figure 3 demonstrates that the friability achieved its minimum value with the simultaneous increase in the levels of PPGS and MCC, with a pronounced effect of PPGS.

### 3.7. Influence of Independent Variables on Disintegration Time of ODMTs

As listed in Table 7, the DT of the produced tablets varied from 30.17 ± 0.81 to 83.11 ± 1.13 s. Additionally, it can be observed that DT accelerated when increasing the level of PPGS (2–10%). Obviously, PPGS show adequate swelling when in contact with water, which results in increasing the swelling pressure inside the tablet and, ultimately, faster disintegration of prepared ODMTs [23]. This finding agrees with Khafagy et al., who argued that increasing the level of PPGS (0–20% *w*/*w*) resulted in faster disintegration of Escitalopram orodispersible tablets. However, in the present study, achieving acceptable DT of ODMTs using a small amount of additive could alleviate the excipients burden in pediatric patients, which is a challenging task. The impact of independent parameters on the DT of produced ODMTs is explained by the empirical model of Equation (9).

(9) $$\mathrm{DT}\;\left(\sec\right)=57.71-3.76{\;X}_{1}-22.56{\;X}_{2}-2.12\;X_{1}X_{2}$$

The ANOVA analysis listed in Table 8 reveals that both independent variables had significant impacts on DT (*p* < 0.0024 for MCC and *p* < 0.0001 for PPGS). In addition, the PPGS level had a pronounced effect on DT, followed by the MCC level, according to the sum of square values (3052.37 for PPGS and 84.98 for MCC). The negative sign of coefficients estimate in Equation (8) (−3.76 for MCC and −22.56 for PPGS) showed that the two variables had positive effects on reducing the DT. Joined with the response surface plot (Figure 3), it could be noticed that the DT achieved its minimum value with the simultaneous increase in the levels of PPGS and MCC with a predominant effect of PPGS. The interaction effect between the two variables (i.e., MCC and PPGS) had no significant effect on the DT (*p* = 0.4243).

### 3.8. Influence of Independent Variables on Tablet Dissolution

**Figure 4:**
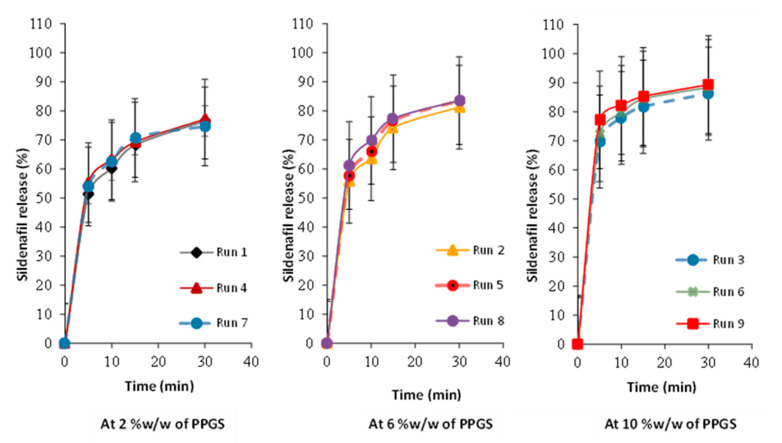
In vitro release profiles of sildenafil ODMTs based on 3^2^ Full-Factorial Design.

Figure 4 displays the release profiles for all formulations. As shown in Table 7, the percentage of sildenafil released after 30 min varied from 76.49 ± 1.15% to 89.31 ± 2.17%. In general, the drug release decreases with increasing DT of ODMTs, as shown in runs 1, 4, and 7, which contain low levels of PPGS (2%), whereas other runs demonstrated an acceptable dissolution profile since they released >80.0% of sildenafil after 30 min. Thus, the dissolution rate of ODMTs was impacted by the level of PPGS. The influence of independent variables on the dissolution of the obtained ODMTs is shown by the empirical model of Equation (10).

(10) $$\mathrm{Sildenafil}\;\mathrm{release}\;\left(\%\right)=83.14+0.5817{\;X}_{1}+5.94{\;X}_{2}+1.23\;X_{1}X_{2}-0.88\;X_{1}{}^{2}-0.53\;X_{2}{}^{2}$$

The ANOVA analysis (Table 8) described that both independent parameters had significant impacts on tablet dissolution (*p* < 0.0333 for MCC and *p* < 0.0001 for PPGS). Further, the PPGS level had a pronounced effect, followed by the MCC level with respect to the values of the sum of square (212.06 for PPGS and 2.30 for MCC). The positive sign of coefficient estimates in Equation (10) (+0.5817 for MCC and +5.94 for PPGS) showed that both variables had positive effects on the percent drug release. Combined with the response surface plot shown in Figure 3, a high level of MCC and PPGS would result in a higher drug release. The rapid release of the drug could be attributed to the fluid bed providing highly porous and low-density granules that rapidly disintegrated and released the incorporated drug [24]. A significant interaction effect (*p* = 0.0031) was found between MCC and PPGS, demonstrating that increasing the concentration of MCC and PPGS resulted in a higher drug release.

### 3.9. Lack-of-Fit Test

The lack-of-fit test is a numerical method applied to explain the residual error and to investigate the validity of functional parts of a suggested model. It matches the residual error with pure error of replication and provides F values for all the suggested models. [25]. As shown in Table 9, it was observed that the calculated F value > the tabulated F value for all the measured responses suggesting a non-significant lack-of-fit (*p* > 0.05).

### 3.10. Formulation Optimization

**Figure 5:**
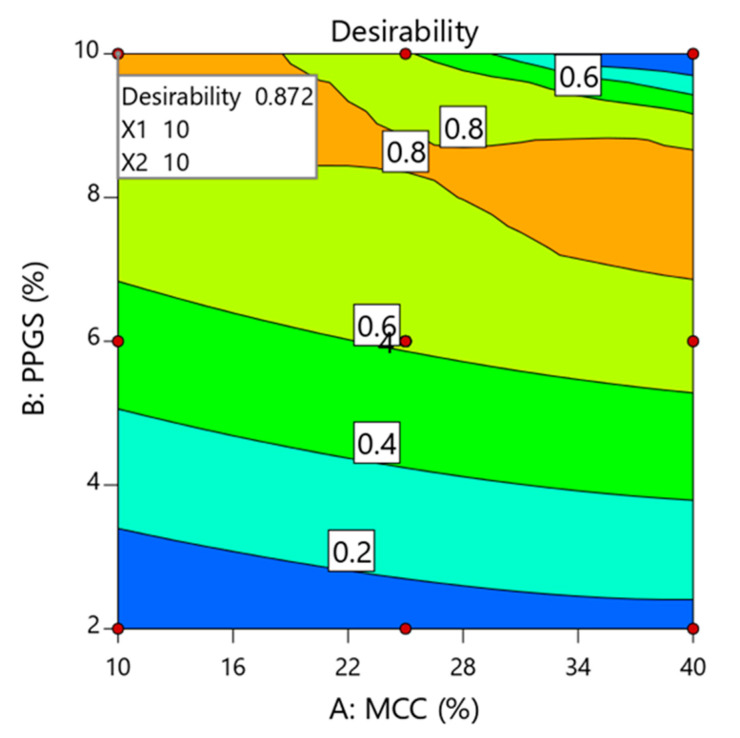
Formulation optimization using global desirability function.

Optimization of ODMTs formulation can be performed by setting the goals for each measured response and then applying the desirability function. As shown in Table 10, crushing strength, friability, DT, and drug release after 30 min were targeted to 5 KP, 0.65%, 35 s, and 85%, respectively. Based on these criteria, the desirability contour plot was produced with a high desirability value of 0.872 (Figure 5). Additionally, optimization using the desirability function indicated that MCC and PPGS at low (10% *w*/*w*) and high (10% *w*/*w*) levels, respectively, were the optimum levels to produce sildenafil ODMTs with the desired quality attributes. Further, the optimized sildenafil ODMTs showed crushing strength of 4.72 ± 0.34 KP, friability of 0.71 ± 0.04%, DT of 39.11 ± 1.03, and sildenafil release of 86.21 ± 2.41% after 30 min, which achieves the USP acceptance criteria for ODMTs. As displayed in Table 11, the prediction error was calculated using predicted and observed values, and the results were found to be within the acceptable values (±5%), thus assuring the validity of the experimental design.

## 4. Conclusions

The present research shows the successful development and optimization of sildenafil ODMTs for the treatment of PAH in pediatric patients with the application of the DoE approach and fluid bed granulation method. A 3^2^ full-factorial design was performed to develop mathematical models describing the relationships between independent formulation variables (levels of MCC and PPGS) and dependent responses of developed sildenafil ODMTs. The ANOVA analysis showed that the levels of MCC and PPGS had a significant effect on the CQAs of the developed sildenafil ODMTs, with the strongest effect of PPGS. Optimization by desirability function indicated that MCC and PPGS at low (10% *w*/*w*) and high (10% *w*/*w*) levels, respectively, were the optimum levels to develop sildenafil ODMTs with the desired quality attributes. The optimized formula of sildenafil ODMTs achieved the desired quality attributes in terms of crushing strength (4.72 ± 0.34 KP), friability (0.71 ± 0.04%), DT (39.11 ± 1.03 s), and sildenafil release after 30 min (86.21 ± 2.41%). Ultimately, a new oral formulation of sildenafil citrate has been successfully developed for the treatment of pulmonary hypertension in pediatric patients. Moreover, at lower drug loadings, the fluid bed granulation method could produce ODMTs with more acceptable quality than previously reported mini-tablets produced from DC and high-shear granulation methods using less amount of excipients. This results in decreasing the excipients burden in pediatric patients as well as improving the dosing flexibility in pediatric clinics. Therefore, this work provides a framework for developing ODMTs with acceptable quality attributes using the fluid bed granulation process.

## Figures and Tables

**Table 1:** QTPP and CQAs of sildenafil MODTs.

QTPP Element	Target	CQAs	Justification
Dosage form	Orodispersible mini-tablets	Breaking force	Hard enough
Appearance and size	Uncoated mini-tablets (round, 3 mm in diameter)	Friability	<1%
Strength Route of administration Proposed indications	5 mg/mini-tablet Oral Pediatric pulmonary hypertension	Disintegration time Drug release -	<60 s Not less than 80% in 30 min -

**Table 2:** A 3^2^ full-factorial experimental design.

Run	MCC (% *w*/*w*)	PPGS (% *w*/*w*)
1	10	2
2	10	6
3	10	10
4	25	2
5	25	6
6	25	10
7	40	2
8	40	6
9	40	10

**Table 3:** Formulation used in the preparation of MODTs.

Ingredients	Function	% *w*/*w*	Quantity (mg)
Sildenafil citrate	Drug	33.33	5
Microcrystalline cellulose PH-105	Filler	10–40	1.5–6
Partially pre-gelatinized starch	Binder/Disintegrant	2–10	0.3–1.5
D-mannitol	Filler	Up to 100	Up to 15
Sodium saccharin	Sweating agent	1	0.15
FDA-approved flavor	Flavor	q.s.	q.s.
Sodium stearyl fumarate	Hydrophilic lubricant	1	0.15
Total	-	100	15

**Table 4:** Summary of model fitting and statistical analysis.

Responses	Suggested Model	*p*-Value	R^2^	Adjusted R^2^	Predicted R^2^	Adequate Precision
Y_1_:D_50_	Linear	<0.0001	0.9094	0.8893	0.2619	17.396
Y_2_: Bulk density	Linear	0.0004	0.8293	0.7914	0.7442	14.861
Y_3_: Angle of repose	Linear	<0.0001	0.8810	0.8545	0.8295	17.775
Y_4_: Crushing strength	Linear	<0.0001	0.9802	0.9758	0.9658	41.670
Y_5_: Friability	Quadratic	<0.0001	0.9846	0.9718	0.9362	26.967
Y_6_: Disintegration time	2FI	<0.0001	0.9918	0.9887	0.9716	50.402
Y_7_: Percent release after 30 min	Quadratic	<0.0001	0.9929	0.9869	0.9381	39.195

**Table 5:** Measured response results of prepared granules (mean ± SD).

Formula	d_50_ (µm ± SD)	Bulk Density (g/mL ± SD)	Tapped Density (g/mL ± SD)	Carr’s Index %	Flow Character according to USP	Angle of Repose (Degree ± SD)
1	106.13 ± 0.35	0.213 ± 0.014	0.251 ± 0.016	15.13	Good	33.21 ± 0.321
2	124.71 ± 0.32	0.231 ± 0.006	0.2626 ± 0.009	11.83	Good	31.49 ± 0.423
3	162.45 ± 0.21	0.252 ± 0.032	0.276 ± 0.037	8.69	Excellent	28.72 ± 0.127
4	111.56 ± 0.26	0.243 ± 0.034	0.281 ± 0.031	13.5	Good	32.46 ± 0.615
5	131.23 ± 0.21	0.273 ± 0.007	0.313 ± 0.009	12.77	Good	30.54 ± 0.247
6	159.58 ± 0.65	0.301 ± 0.033	0.323 ± 0.041	6.81	Excellent	27.84 ± 0.442
7	116.24 ± 0.24	0.287 ± 0.008	0.328 ± 0.008	12.5	Good	31.77 ± 0.361
8	134.85 ± 0.32	0.301 ± 0.047	0.326 ± 0.062	7.66	Excellent	28.11 ± 0.431
9	161.74 ± 0.45	0.322 ± 0.019	0.337 ± 0.017	4.45	Excellent	26.81 ± 0.392

**Table 6:** ANOVA analysis of granules measured responses.

Variables	Coefficient Estimate	Sum of Squares	Standard Error	F-Value	*p*-Value	95% CI Low	95% CI High
**Y_1_: D_50_ (Linear model)**							
**Intercept**	136.95	-	1.87	-	-	132.71	141.18
**X_1_**	3.26	63.64	2.65	1.51	**0.2503**	−2.74	9.25
**X_2_**	24.97	2742.00	2.65	88.81	**<0.0001**	18.98	30.97
**Y_2_: Bulk density (Linear model)**							
**Intercept**	0.2759	-	0.0045	-	-	0.2658	0.2961
**X_1_**	0.0357	0.0076	0.0063	31.68	**0.0003**	0.0213	0.0500
**X_2_**	0.0220	0.0029	0.0063	12.05	**0.007**	0.0077	0.0363
**Y_3_: Angle of repose (Linear model)**							
**Intercept**	29.95	-	0.2252	-	-	29.44	30.46
**X_1_**	−1.12	7.55	0.3185	12.40	**0.0065**	−1.84	−0.4012
**X_2_**	−2.35	32.99	0.3185	54.22	**<0.0001**	−3.07	−1.62

X_1_ and X_2_ are MCC and PPGS levels, respectively.

**Table 7:** Measured response results of prepared ODMTs (mean ± SD).

Run	Weight (mg ± SD)	Drug Content (% ± SD)	Crushing Strength (KP ± SD)	Friability (% ± SD)	Disintegration Time (S ± SD)	%Release at 30 min (% ± SD)
1	14.86 ± 1.31	100.32 ± 1.25	2.86 ± 0.61	1.22 ± 0.03	83.11 ± 1.13	76.49 ± 1.15
2	14.93 ± 1.27	99.13 ± 1.41	3.93 ± 0.14	0.94 ± 0.06	60.42 ± 1.33	81.24 ± 1.14
3	15.91 ± 1.21	101.33 ± 1.22	4.71 ± 0.61	0.74 ± 0.02	43.15 ± 1.28	86.24 ± 1.13
4	14.98 ± 1.05	100.66 ± 2.11	2.93 ± 0.33	1.18 ± 0.01	79.34 ± 1.63	77.11 ± 2.36
5	14.89 ± 1.15	98.89 ± 1.71	4.15 ± 0.28	0.86 ± 0.05	57.62 ± 1.61	83.47 ± 1.82
6	14.95 ± 1.29	101.56 ± 0.98	5.11 ± 0.71	0.69 ± 0.03	32.41 ± 1.32	88.35 ± 2.41
7	15.25 ± 0.88	100.88 ± 1.63	3.34 ± 0.31	1.07 ± 0.02	78.61 ± 2.18	74.63 ± 2.84
8	15.13 ± 0.74	98.76 ± 2.01	4.32 ± 0.39	0.81 ± 0.01	55.32 ± 0.57	83.52 ± 1.21
9	15.23 ± 0.48	100.93 ± 0.76	5.31 ± 0.26	0.65 ± 0.03	30.17 ± 0.81	89.31 ± 2.17

**Table 8:** ANOVA analysis of ODMTs measured responses.

Variables	Coefficient Estimate	Sum of Squares	Standard Error	F-Value	*p*-Value	95% CI low	95% CI High
**Y_4_:** **Crushing strength (Linear model)**							
**Intercept**	4.11	-	0.0345	-	-	4.03	4.19
**X_1_**	0.245	0.3601	0.0488	25.22	**0.0007**	0.1346	0.3554
**X_2_**	1.00	6.00	0.0488	420.10	**<0.0001**	0.8896	1.11
**Y_5_:** **Friability (Quadratic model)**							
**Intercept**	0.8462	-	0.0140	-	-	0.8119	0.8806
**X_1_**	−0.0617	0.0228	0.0126	24.11	**0.0027**	−0.0924	−0.0309
**X_2_**	−0.2317	0.3220	0.0154	340.21	**<0.0001**	−0.2624	−0.2009
**X_1_ X_2_**	0.0115	0.0009	0.0188	0.9508	**0.3672**	−0.0226	−0.0526
**Y_6_:** **Disintegration time (2FI model)**							
**Intercept**	57.71	-	0.5222	-	-	56.50	58.91
**X_1_**	−3.76	84.98	0.7384	25.97	**0.0009**	−5.47	−2.06
**X_2_**	−22.56	3052.37	0.7384	932.92	**<0.0001**	−24.26	−20.85
**X_1_ X_2_**	−2.12	17.98	0.9044	5.49	**0.0471**	−4.21	−0.0344
**Y_7_:** **Percent release at 30 min (Quadratic model)**							
**Intercept**	83.14	-	0.2364	-	-	82.56	83.72
**X_1_**	0.5817	2.03	0.2114	7.57	**0.0333**	0.0643	1.10
**X_2_**	5.94	212.06	0.2114	790.49	**<0.0001**	5.43	6.46
**X_1_ X_2_**	1.23	6.08	0.2590	22.65	**0.0031**	0.5988	1.87

X_1_ and X_2_ are MCC and PPGS levels, respectively, X_1_X_2_ is the effect of interaction.

**Table 9:** Lack-of-fit test of measured responses.

Response	F-Value	*p*-Value	Comment
d_50_	0.7929	0.6313	Not significant
Bulk density	0.9518	0.5638	Not significant
Flowability	0.1755	0.9656	Not significant
Crushing strength	1.77	0.3419	Not significant
Friability	0.7341	0.5972	Not significant
Disintegration time	1.39	0.4180	Not significant
Percent release after 30 min	4.99	0.1098	Not significant

**Table 10:** The constraints adopted for developed design space.

Variables	Target	Range	Weight	Importance Co-Efficient
**Input**				
MCC	In range	10–40% *w*/*w*	1	NA
PPGS	In range	2–10% *w*/*w*	1	NA
**Output**				
Crushing strength (KP)	5	2.86–5.31		+++++
Friability (%)	0.65	0.65–1.22	1	+++
Disintegration time (s)	35	30.17–83.11	1	+++++
Percent release after 30 min (%)	85	74.63–89.31	1	+++

**Table 11:** Predicted and experimental values of optimized run with their relative errors.

Variables	Value		
MCC (% *w*/*w*)	10		
PPGS (% *w*/*w*)	10		
Overall desirability = 0.872			
**Responses**	**Predicted values**	Experimental values *	**Prediction error (%)**
Crushing strength (KP)	4.86	4.72 ± 0.34	2.88
Friability (%)	0.74	0.71 ± 0.04	4.22
Disintegration time (s)	41.03	39.11 ± 1.03	4.67
Percent release after 30 min (%)	85.85	86.21 ± 2.41	−4.88

* Experimental (actual) values are presented as mean ± SD.

## Data Availability

The datasets used and/or analyzed in the current study are available from the corresponding author upon reasonable request.
